# Acupuncture Therapy plus Hyaluronic Acid Injection for Knee Osteoarthritis: A Meta-Analysis of Randomized Controlled Trials

**DOI:** 10.1155/2020/4034105

**Published:** 2020-01-07

**Authors:** Yang Zheng, Xiangdong Duan, Shangfeng Qi, Haibo Hu, Mengran Wang, Conglin Ren, Haipeng Xu, Renfu Quan

**Affiliations:** ^1^Clinical Medical College, Zhejiang Chinese Medical University, Hangzhou 310051, China; ^2^Department of Orthopedics, Shandong Provincial Hospital of Traditional Chinese Medicine, Jinan 250014, China; ^3^Clinical Medical College, Shandong University of Traditional Chinese Medicine, Jinan 250014, China; ^4^Department of Orthopedics, Xiaoshan Traditional Chinese Medical Hospital, Hangzhou 311200, China

## Abstract

**Objective:**

This meta-analysis aimed to investigate the effectiveness of acupuncture therapy plus hyaluronic acid injection versus hyaluronic acid injection alone for patients with knee osteoarthritis.

**Methods:**

Relevant randomized controlled trials that compared the combined effect of acupuncture therapy and hyaluronic acid injection with hyaluronic acid injection alone for knee osteoarthritis patients were included. 10 studies were included in this meta-analysis, and the relative risk (RR) and weight mean difference (MD) with 95% CI for the Lysholm knee score (LKSS), visual analogue scale (VAS), and effective rate (ER) were evaluated by using RevMan 5.3 software. Besides, the bias assessment of the included studies was evaluated using the Cochrane risk of bias tool, and the GRADE (Grading of Recommendations, Assessment Development, and Evaluation) system was applied to assess the overall quality of the evidence.

**Results:**

A total of 10 studies involving 998 participants were included in this study. Compared to hyaluronic acid injection alone, the combined therapy significantly reduced pain on the visual analogue scale (VAS) and improved the ER and knee function on the Lysholm knee score (LKSS). Of these, the pooled LKSS (MD = 8.09, 95% CI = [7.02, 9.16], *p* < 0.00001, 7 studies) and ER (RR = 1.23, 95% CI 1.15 to 1.31, *p* < 0.00001, 8 studies) suggested that combination therapy yielded a significantly higher ER and improved the LKSS scores to a greater degree than hyaluronic acid injection alone in patients with KOA. The VAS (MD = −1.39, 95% CI = [−1.99, −0.79], *p* < 0.00001, 7 studies) showed that the combined therapy significantly reduced pain than hyaluronic acid injection alone. The quality of evidence for the main outcomes was from very low to low according to the GRADE system.

**Conclusion:**

Current evidence suggests that acupuncture therapy combined with hyaluronic acid injection is more effective in alleviating pain, improving the ER and knee function compared with hyaluronic acid injection alone. However, considering the low quality, small size, and high risk of the studies identified in this meta-analysis, more higher methodological quality, rigorously designed randomized controlled trials with large sample sizes are needed to confirm the results.

## 1. Introduction

Knee osteoarthritis is a highly common chronic degenerative disease in middle-aged and elderly people, with more female patients than males, which not only seriously affects joint function and the quality of life, but also becomes a serious public health problem worldwide [1–3]. With accelerating aging process of the social population, the morbidity of degenerative osteoarthritis in the world has increased significantly [4]. KOA is generally thought to be caused by a variety of pathogenic factors such as age, gender, weight, trauma, and genetics and characterized by joint pain and dysfunction, with progressive intraarticular cartilage and subchondral bone injury, synovitis, osteophyte formation, and joint cavity reduction [5, 6]. Current conservative treatment strategies for KOA include chondroitin sulfate, massage, extracorporeal shock wave, acupuncture, exercise therapy, hydrotherapy, ozone therapy, glucocorticoids, hyaluronic acid, nonspecific anti-inflammatory drugs, reducing body mass, and knee strength training [7–10].

Among these conservative therapies, acupuncture, known as an ancient, complementary, and alternative therapeutic technique [11], has been empirically practiced and improved over thousands of years in Asian countries and verified to be the most effective and popular therapies in treating the pain symptoms and functional disability of patients with KOA based on integral concepts and syndrome differentiation of the TCM system. The effectiveness of acupuncture for treating many diseases has been verified in a series of high-quality clinical trials [12–14]. According to the ACR guidelines, acupuncture is conditionally recommended for KOA for its pain relief, affordability, and safety [15–18]. In addition, hyaluronic acid products, commonly used as alternative intraarticular injection and recommended by the Food and Drug Administration for treatment of KOA in 1997 [19], have the function of viscoinduction properties and increasing intraarticular lubrication, inhibiting inflammatory mediators and promoting repair of cartilage to delay the progression of KOA [20, 21]. Altman et al. [22] has reported that HA injections are generally well tolerated, provide a longer duration of symptomatic relief, and improve knee function for patients with knee OA.

However, acupuncture therapy combined with HA injection is also a common method for KOA treatment, but whether the combined application of these two methods is better than HA alone is still lacking of systematic evaluation. Therefore, we conducted the present meta-analysis of randomized controlled trials (RCTs) to assess the efficacy of acupuncture therapy plus HA injection compared with that of HA alone in patients with knee OA.

## 2. Materials and Methods

### 2.1. Search Strategy and Selection Criteria

Comprehensive electronic and manual searches were independently retrieved in 7 databases by two reviewers: PubMed, the Cochrane Library, EMBASE, China National Knowledge Infrastructure (CNKI), Wanfang Database, Chinese Scientific Journal Database (VIP database), and Chinese Biomedical Literature Database (Sinomed), from their inception to 2019. The search terms consisted of four parts: (i) Osteoarthritis, Knee (Mesh); Knee Osteoarthritides; Knee Osteoarthritis; Osteoarthritides, Knee; Osteoarthritis Of Knee; Knee, Osteoarthritis Of; Knees, Osteoarthritis Of; Osteoarthritis Of Knees. (ii) Acupuncture Therapy (Mesh); Acupuncture; Needle Acupuncture; Acupuncture Treatment; Manual Acupuncture; Acupuncture Treatment; Acupotomy; Acupotomies; Acupuncture Points; Electroacupuncture; Warm Acupuncture. (iii) Hyaluronic Acid (Mesh); Acid, Hyaluronic; Amo Vitrax; Vitrax, Amo; Biolon; Etamucine; Hyaluronan; Hyvisc; Luronit; Sodium Hyaluronate; Hyaluronate, Sodium; Hyaluronate Sodium; Amvisc; Healon. (iv) randomized controlled trial (Mesh); randomized; randomly; random. The detailed search strategies are presented in [Supplementary-material supplementary-material-1]. Besides, we also searched the references cited in the searched studies so as not to leave out the relevant eligible trials.

### 2.2. Study Selection

#### 2.2.1. Inclusion Criteria for Studies

1. Patients: included patients who met the diagnostic criteria of KOA
2. Study design: the trials had to be randomized controlled trials (RCTs) that compared acupuncture therapy plus hyaluronic acid injection with hyaluronic acid injection alone
3. Outcome measures: primary endpoints are the Lysholm knee score (LKSS) and visual analogue scale (VAS); secondary endpoint is clinical effective rate (ER)
4. Studies were published in English or Chinese, with the full text available

#### 2.2.2. Exclusion Criteria for Studies

1. If the studies were not RCTs
2. Enrolling participants with severe physical or mental disease
3. Duplicate publications, or incomplete data
4. Participants in the intervention or control group underwent other therapies such as surgery, western medicine, or traditional Chinese medicine

### 2.3. Data Extraction and Management

Two separate reviewers independently extracted the data from the identified studies based on a unified form according to the predetermined criteria. For each study, first author's name, the year of publication, sample size, sex, age, duration, details of treatment and control procedures, acupuncture treatment duration, total period, main results, and outcome measures were recorded. Disagreements were resolved through discussion. A third reviewer was consulted if discrepancies persisted. Insufficient information about some of the included trials was obtained by contacting the authors by e-mail.

### 2.4. Bias Assessment of the Included Studies

Risk of bias was assessed independently by two reviewers using the Cochrane risk of bias tool for the following seven criteria: random sequence generation (selection bias), allocation concealment (selection bias), blinding of participants and personnel (performance bias), blinding of outcome assessment (detection bias), incomplete outcome data (attrition bias), selective reporting (reporting bias), and other bias. The judging criteria were as follows: “Yes” for low risk, “Unclear” for unclear, and “No” for high risk. A third reviewer appraised the discrepancy and made the final decision regarding the ratings.

### 2.5. Statistical Analyses

The weighted mean difference (MD) with 95% CI was applied for continuous data, and the pooled relative risk (RR) and 95% confidence interval (CI) was reported for the clinical efficacy rate (ER). The potential heterogeneity among the included studies was assessed by the chi-square test and the inconsistency index statistic (*I*^2^), and the fixed-effects model was applied to analyze the data without obvious heterogeneity (*p* > 0.1 and *I*^2^ < 50%); otherwise, for *p* > 0.1 and *I*^2^> 50%, it was considered that the trials included were heterogeneous and a random-effects model was used, and we would try to explore the potential sources of it by sensitivity analysis. In view of different acupuncture therapy techniques, subgroup analyses were further performed. Additionally, the potential publication bias was assessed by applying the funnel plot if the number of studies for an outcome was adequate (*n* ≥ 10).

### 2.6. Quality of Evidence

We used the GRADE (Grading of Recommendations Assessment, Development, and Evaluation) working group method to appraise the quality of evidence, and the GRADEpro software (version 3.6 for Windows, GRADE Working Group) was applied.

## 3. Results

### 3.1. Literature Selection

**Figure 1:**
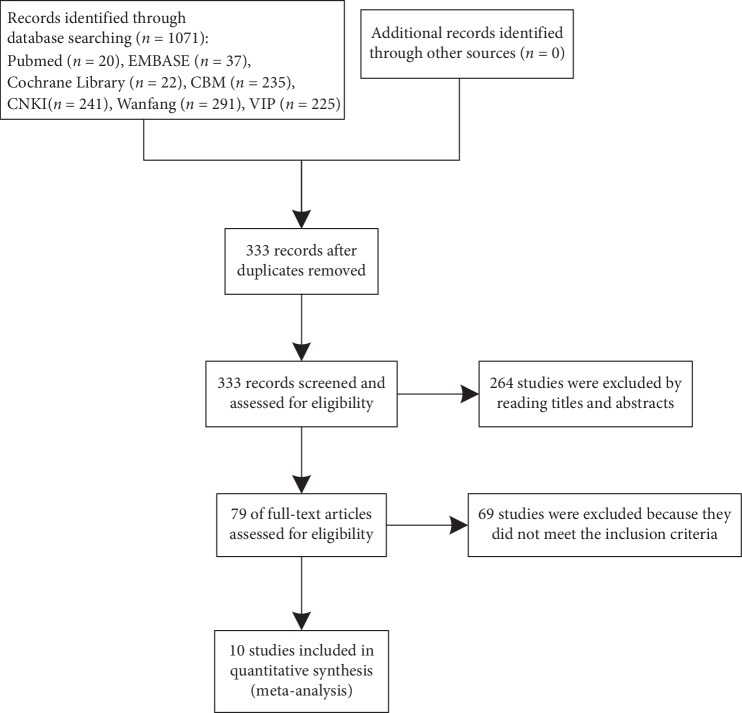
Flow chart for the studies' screening process.

We identified 1071 studies based on our retrieval strategies, of which 992 studies were removed by screening the titles and abstracts since they were not qualified according to the predefined inclusion criteria. A further 69 studies were excluded according to the exclusion criteria through reviewing the full texts. Finally, ten studies were included in the meta-analysis.  Figure 1 describes the detailed selection process for relevant studies.

### 3.2. Characteristics of Included Literature

In the 10 eligible randomized controlled trials involving 998 patients (470 men and 528 women), 501 received acupuncture therapy plus hyaluronic acid injection in the intervention group and 497 received hyaluronic acid injection in the control group. Among them, the age range of participants adopted into the meta-analysis ranged from 24 to 86. All of the included studies showed no significant difference with the baseline, and all of that were conducted in China and published in Chinese. These included trials were all published between 2012 and 2018.

Among the intervention groups, the main acupuncture techniques applied were warm acupuncture (WA, *n* = 4) [23–26], acupotomy (AT, *n* = 3) [27–29], manual acupuncture (MA, *n* = 2) [30, 31], and AT plus WA (*n* = 1) [32]. Additionally, the most commonly adopted 6 acupuncture points were Neixiyan (Ex-LE4), Xuehai (SP10), Yanglingquan (GB34), Zusanli (ST36), Weizhong (BL40), and Shenshu (BL23). Moreover, each treatment time ranged from about 15 to 30 minutes and treatment regimens varied from one to seven sessions per week. All patients in both groups received HA injection in the involved knee joint with 2 ml HA solution (*n* = 8), 2.5 ml HA solution (*n* = 1), and 3 ml HA solution (*n* = 1), and the treatment period ranged from 3 to 5 weeks and the included trials had showed follow-up periods ranging from 1 month to 1 year [27–29, 32]. The specific characteristics of these included studies are shown in Table 1.

### 3.3. Methodological Quality of Included Studies

**Figure 2:**
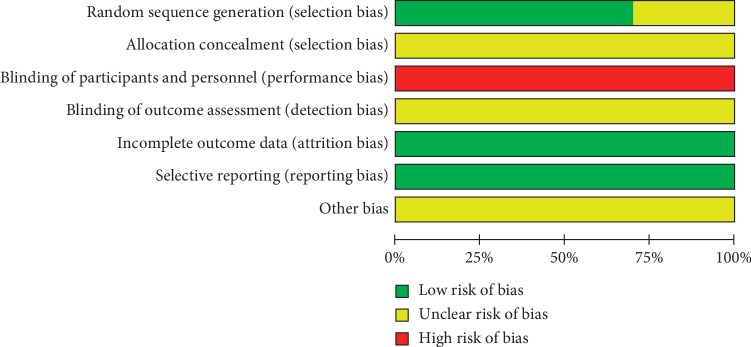
Risk of bias.

**Figure 3:**
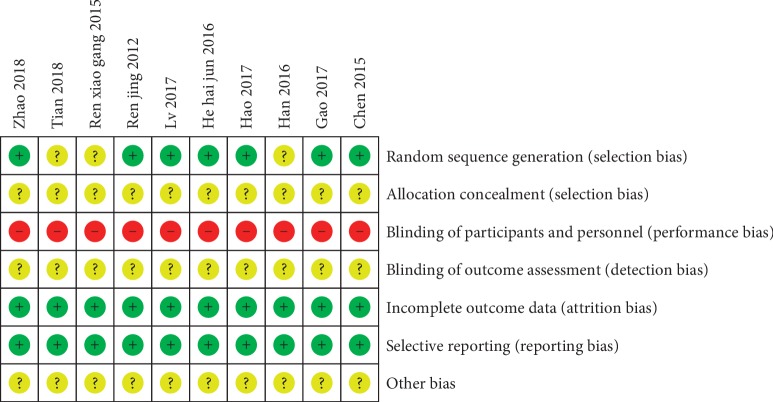
Risk of bias summary.

The risk of bias assessment for the included studies is shown in Figures 2 and 3. In the generation of randomization sequence, patients were randomized by using the random number table in 6 studies [23, 26, 28–30, 32] and by applying the SAS software in one study [31] and the remaining 3 studies [24, 25, 27] only mentioned “random” or “randomization” without describing the explicit randomization technique. Noticeably, no studies described allocation concealment and method of blinding in a detailed way. Given the characteristics of the design between the intervention and control groups, it was not possible to blind participants and personnel, thus all studies were judged to be at high risk of bias. Additionally, unclear risk of bias was observed across all studies for detection bias and low risk of bias for attrition and reporting bias.

### 3.4. Results of Meta-Analysis

#### 3.4.1. Lysholm Scores (LKSS)

**Figure 4:**
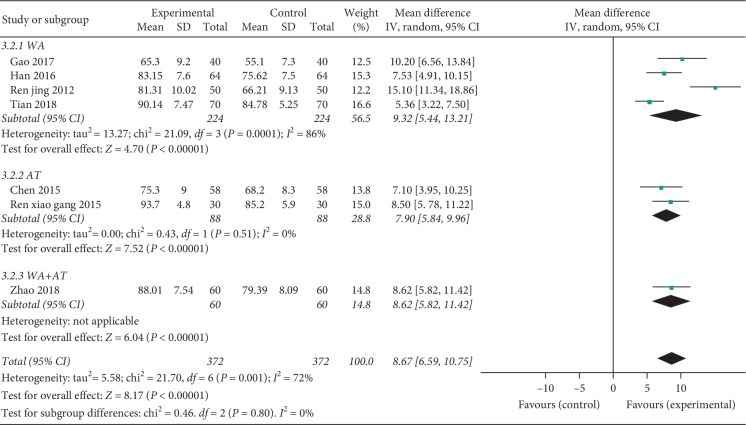
Forest plot of combination group versus control group: Lysholm scores (LKSS).

Seven RCTs (including 744 patients) assessed the improvement of combination therapy on LKSS for KOA versus the control group [23–27, 29, 32]. The meta-analysis indicated superior effects of combination therapy on LKSS improvement (MD = 8.09, 95% CI = [7.02, 9.16], *p* < 0.00001). However, a heterogeneity test (*p* for heterogeneity = 0.001, *I*^2^ = 72%) indicated that there was moderate statistical heterogeneity between studies; therefore, a random-effects model was used and we conducted subgroup analyses based on distinct acupuncture techniques: warm acupuncture (WA), acupotomy (AT), and acupotomy plus warm acupuncture (WA + AT). Subgroup analyses showed remarkably increases of the Lysholm scores in the WA combination therapy with severe heterogeneity (MD = 8.05, 95% CI = [6.65, 9.44], *p* < 0.00001, *p* for heterogeneity = 0.0001, *p* = 86%); AT combined with HA injection similarly enhances Lysholm scores relative to the control group treated with HA injection alone (MD = 7.90, 95% CI = [5.84, 9.96], *p* < 0.00001) with no heterogeneity (*p* for heterogeneity = 0.51, *I*^2^ = 0%) and AT plus WA (MD = 8.62, 95% CI = [5.82, 11.42], *p* < 0.00001)(Figure 4).

#### 3.4.2. Visual Analogue Scale (VAS)

**Figure 5:**
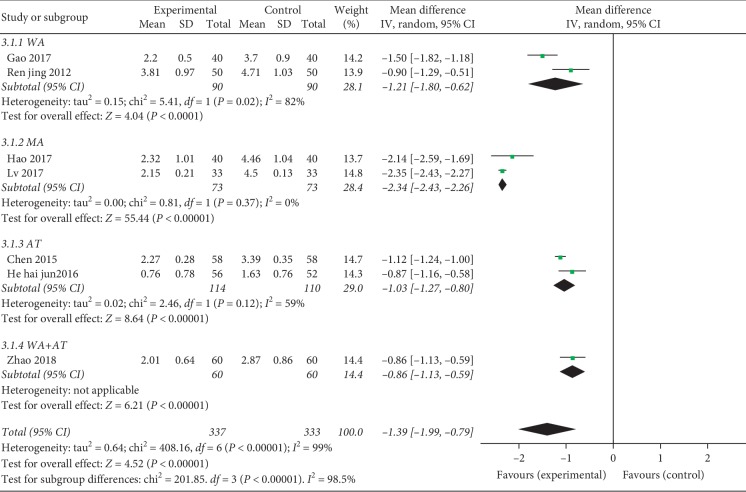
Forest plot of combination group versus control group: visual analogue scale (VAS).

There were 7 studies reporting VAS for KOA [23, 26, 28–32] (including 670 patients). The results of the meta-analysis showed favorable effects of combination therapy on pain relief compared with HA injection alone (MD = −1.39, 95% CI = [−1.99, −0.79], *p* < 0.00001) (Figure 2). A heterogeneity test (*p* for heterogeneity <0.00001, *I*^2^ = 99%) indicated that there was severe statistical heterogeneity between studies; therefore, a random-effects model was used and subgroup analysis by the acupuncture techniques was further conducted to explore the potential source of heterogeneity. For WA, MD = −1.21, 95% CI = [−1.80, −0.62], *p* < 0.0001, *p* for heterogeneity = 0.02, and *I*^2^ = 82%; for MA, MD = −2.34, 95% CI = [−2.43, −2.26], *p* < 0.00001, *p* for heterogeneity = 0.37, and *I*^2^ = 0%; for AT, MD = −1.03, 95% CI = [−1.27, −0.80], *p* < 0.00001, *p* for heterogeneity = 0.12, and I^2^ = 59%; and for WA plus AT, MD = −0.86, 95% CI = [−1.13, −0.59], and *p* < 0.00001 (Figure 5).

#### 3.4.3. Clinical Efficacy Rate (ER)

**Figure 6:**
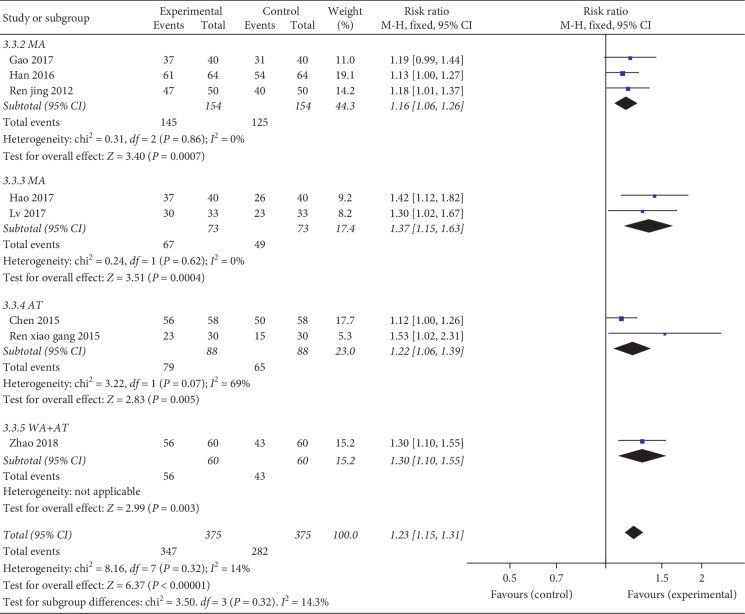
Forest plot of combination group versus control group: efficacy rate (ER).

Among all 10 included studies, 8 RCTs [23, 24, 26, 27, 29–32] (including 750 patients) reported the clinical effective rate of the patients in the two groups. There was no significant heterogeneity (*p*=0.32, *I*^2^ = 14%) among these RCTs; thus, a fixed-effect model was used to calculate the combined RR and 95% CI, and our pooled results showed favorable effects of acupuncture therapy plus hyaluronic acid injection on effective rate (RR = 1.23, 95% = [CI 1.15, 1.31], and *p* < 0.00001). According to different acupuncture therapy technologies, subgroup analyses were conducted. For warm acupuncture, RR = 1.16, 95% CI = [1.06, 1.26], *p* < 0.001, *p* for heterogeneity = 0.86, and *I*^2^ = 0%; for manual acupuncture, RR = 1.37, 95% CI = [1.15, 1.63], *p* < 0.001, *p* for heterogeneity = 0.62, and *I*^2^ = 0%; for acupotomy, RR = 1.22, 95% CI = [1.06, 1.39], *p*=0.005, *p* for heterogeneity = 0.07, and *I*^2^ = 69%; and acupotomy plus warm acupuncture, (RR = 1.30, 95% CI = [1.10, 1.55], and *p*=0.003) combined therapy significantly improved clinical effectiveness compared with hyaluronic acid injection only (Figure 6).

### 3.5. Quality of Evidence

The GRADEpro was utilized to evaluate the quality of evidence for the meta-analysis. We assessed the Lysholm knee score (LKSS), the visual analogue scale (VAS), and the clinical effective rate (ER). Details are shown in the GRADE evidence profile, and the summary of findings is shown in Table 2.

The results show that the quality of evidence was from low for the assessment of LKSS because 3 studies mentioned “random” or “randomization” without describing the detailed randomization technique. Besides, all of the studies did not specifically describe the method of allocation concealment, there was a high risk of performance bias across the studies due to the difficulty of blinding to participants and personnel, and the number of adverse events of included studies was not sufficiently reported, thus the quality of evidence of publication bias was downgraded. For VAS, the quality of evidence was very low because of the selection and performance bias, the significant heterogeneity, and the insufficient adverse events. For ER, the quality of evidence was low due to the selection and performance bias and the insufficient adverse events.

### 3.6. Preferred Acupuncture Points

We collected the data related to the acupuncture points selected from the enrolled studies and found that six acupuncture points were most frequently used to treat KOA, namely, Neixiyan (Ex-LE4), Xuehai (SP10), Yanglingquan (GB34), Zusanli (ST36), Weizhong (BL40), and Shenshu (BL23). The overall acupuncture points of these included studies are shown in [Supplementary-material supplementary-material-1].

### 3.7. Adverse Events

Of the 10 studies, 2 studies [23, 31] reported that no serious adverse events were observed during the treatment, one study [30] reported that there were injection site pain (2 cases in the experimental group and 1 case in the control group) and knee discomfort (1 case in the experimental group), and 5 studies (50%) had no mention of the occurrence of adverse events. The remaining 2 studies [27, 28] (20%) stated that no AEs occurred.

## 4. Discussion

### 4.1. Principal Findings

To our knowledge, this is the first meta-analysis regarding published evidence to investigate the effectiveness of the combination of acupuncture therapy plus hyaluronic acid injection versus hyaluronic acid injection alone for treating KOA patients. Acupuncture therapy and hyaluronic acid injection as routine therapies are commonly used to treat patients with knee osteoarthritis based on traditional Chinese medicine or modern Western medicine. However, no strong evidence provided by previous researches has demonstrated whether the combination therapy has a more positive impact on KOA than hyaluronic acid injection alone. Therefore, in the present study, a meta-analysis of relevant RCTs was designed to provide a reliable quantitative evaluation of existing evidence on the effectiveness of acupuncture therapy and hyaluronic acid injection for the treatment of KOA.

To comprehensively assess the value of acupuncture therapy plus hyaluronic acid injection, data on Lysholm knee score (LKSS), visual analogue scale (VAS), and clinical effective rate (ER) were chosen to evaluate for improvements in multiple dimensions among patients with KOA. Based on our meta-analysis, the pooled data of LKSS and VAS showed that the combined application of acupuncture therapy and HA injection improved knee function and alleviated pain to a significantly greater degree (MD = 8.09, 95% CI = [7.02, 9.16], *p* < 0.00001; MD = −1.39, 95% CI = [−1.99, −0.79], *p* < 0.00001, respectively), than HA alone. The estimated effect sizes of ER suggested that the effective rate was significantly better in the combination therapy group than in the HA injection alone group (RR = 1.23, 95% CI 1.15 to 1.31, *p* < 0.00001).

Overall, the effectiveness of acupuncture therapy plus HA injection for patients with KOA was confirmed by this meta-analysis. Regarding acupuncture points, we summarised the most commonly adopted six acupuncture points based on the included studies and thereby provide recommendations for clinical and research settings.

### 4.2. Limitations of These Studies

The current meta-analysis has a number of limitations that must be acknowledged, as shown in the following:

1. No studies enrolled in this meta-analysis had mentioned allocation concealment, blinding of participants and personnel, and blinding of outcome assessments.
2. Most of the included studies in the meta-analysis had a relatively small sample size which limited the dependability of the pooled results, and the therapeutic effect may be overestimated in smaller studies compared with larger sample studies.
3. The studies included in this meta-analysis were all conducted in China where acupuncture is well endowed, widely researched and practiced, and all published in Chinese language, but KOA is a worldwide disease. Besides, the risk of publication bias with the funnel plot cannot be evaluated because of limited number of trials. Therefore, the results might have language and reporting bias.
4. The duration of therapy in the majority of the included studies lasted 4-5 weeks, and only four studies had mentioned the follow-up period ranging from 3 months to 1 year. However, KOA is a chronic disease which should include adequate duration of therapy and follow-up periods in the observations of researches.
5. Our review may be affected by the high heterogeneity, and AEs were not sufficiently reported.

## 5. Conclusion

This meta-analysis of 10 randomized controlled trials provides evidence confirming that acupuncture therapy plus hyaluronic acid injection can improve the ER and LKSS and reduce VAS in patients with KOA compared with hyaluronic acid injection alone. However, the findings should be interpreted cautiously because of the poor methodological quality and heterogeneity of the included studies. Consequently, further rigorously designed and higher quality trials with a larger sample size are necessary for overcoming the limitations of the current study and enhancing the strength of evidence.

## Figures and Tables

**Table 1:** Features of the included studies.

First author (year)	Sample size	Sex (M : F)	Age (year) (mean ± SD)	Duration (mean ± SD)(or range)	Intervention (combination therapy)	Control group (HA alone)	Outcome measures	Follow-up
Chen Y. (2015)	116	T: 26 : 32 C: 28 : 30	T: 56.1 ± 4.8 C: 55.0 ± 4.6	T: 3.7 ± 2.0 y C: 3.9 ± 2.1 y	AT + HA 1 time per week (4 weeks)	2 ml 1 time per week (4 weeks)	LKSS, VAS, ER	1 year
Gao Y. (2017)	80	T: 30 : 10 C: 28 : 12	T: 59.2 ± 3.3 C: 60.5 ± 3.9	T: 6.5 ± 0.5 y C: 6.9 ± 0.9 y	WA + HA 1 time per day (15 min, 5 weeks)	2.5 ml 1 time per week (5 weeks)	WOMAC, QOL, VAS, LKSS	5 weeks
Han D. (2016)	128	T: 34 : 3 C: 28 : 36	T:62.4 ± 6.8 C:63.0 ± 6.5	T: 5.83 ± 0.4 y C: 6.02 ± 0.2 y	WA + HA 3 times per week (30 min, 4 weeks)	2 ml 1 time per week (4 weeks)	LKSS, ER	4 weeks
Hao Y. F. (2017)	80	T: 17 : 23 C: 19 : 21	T: 55.91 ± 6.32 C: 56.32 ± 5.92	T: 52.32 ± 46.5 d C: 53.67 ± 45.8 d	MA + HA 1 time per day (30 min, 5 weeks)	2 ml 1 time per week (5 weeks)	VAS, SF-36, ER, WOMAC	5 weeks
He H. J. (2016)	108	T: 12 : 44 C: 10 : 42	T:50.32 ± 14.38 C:54.05 ± 14.07	T: 4 m–10 y C: 3 m–9 y	AT + HA 1 time per week (2 weeks)	3 ml 1 time per week (5 weeks)	HSS, VAS	3 months
Lv L. (2017)	66	I: 16 : 17 C: 18 : 15	I: 44.10 ± 0.18 C: 44.11 ± 0.15	NM	MA + HA 5 times per week (30 min, 4 weeks)	2 ml 1 time per week (4 weeks)	ER, VAS, HSS	1 month
Ren J. (2012)	100	I: 24 : 26 C: 22 : 28	NM	NM	WA + HA 3 times per week (4 weeks)	2 ml 1 time per week (4 weeks)	VAS, LKSS, ER	1 month
Ren X. G. (2015)	60	T: 11 : 19 C: 13 : 17	T: 63.8 ± 9.8 C: 64.1 ± 10.9	T: 3.4 ± 1.4 y C: 3.2 ± 1.4 y	AT + HA 1 time every 2 weeks (4 weeks)	2 ml 1 time per week (4 weeks)	LKSS, ER	6 months
Tian H. J. (2018)	140	T: 34 : 36 C: 32 : 38	T: 59.5 ± 17.5 C: 61.5 ± 20.5	NM	WA + HA NM	2 ml 1 time per week (4 weeks)	LKSS, QOL	1 month
Zhao Z. C. (2018)	120	T: 35 : 25 C: 33 : 27	T: 62.06 ± 4.37 C: 61.23 ± 4.25	T: 6.50 ± 3.04 y C: 6.38 ± 3.14 y	MA + AT + HA 7 times per week (30 min, 4 weeks)	2 ml 1 time per week (4 weeks)	LKSS, VAS, JOA, ER	6 months

AT: acupotomy; WA: warm acupuncture; MA: manual acupuncture; NM: not mentioned; LKSS: Lysholm scores; VAS: visual analogue scale; ER: clinical efficacy rate; SF-36: Medical Outcomes Study 36-Item Short Form Health Survey; WOMAC: The Western Ontario and McMaster Universities Osteoarthritis Index; QOL: the scales of quality of life; JOA: the scales of Japanese Orthopedics Association; HSS: hospital for special surgery.

**Table 2:** The quality of evidence.

Outcomes	Effect		Number of participants (studies)	Quality of the evidence (GRADE)
	Relative effect (95% CI)	Absolute effect (95% CI)		
LKSS	—	MD 8.67 higher (6.59 to 10.75 higher)	744 (7 studies)	⊕⊕⊝⊝ low^1,3^
VAS	—	MD 1.39 lower (1.99 to 0.79 lower)	670 (7 studies)	⊕⊝⊝⊝ very low^1,2,3^
ER	RR 1.23 (1.15 to 1.31)	173 more per 1000 (from 113 more to 233 more)	750 (8 studies)	⊕⊕⊝⊝ low^1,2^

^1^High risk of performance bias; one of the studies describe the allocation concealment. ^2^Adverse events were not sufficiently reported. ^3^Significant heterogeneity.

## Supplementary Materials

Supplementary Materials Appendix 1: the retrieval strategy. Appendix 2: acupoints in the included trials.
