# Supplementary material for: Acupuncture Therapy plus Hyaluronic Acid Injection for Knee Osteoarthritis: A Meta-Analysis of Randomized Controlled Trials
Source: Evid Based Complement Alternat Med. 2020 Jan 7;2020:4034105. doi: 10.1155/2020/4034105 (PMC6982364; doi:10.1155/2020/4034105)
Supplement: Supplementary Materials — Appendix 1: the retrieval strategy. Appendix 2: acupoints in the included trials. [file 4034105.f1.docx]

**Appendix 1(Search Strategy of 7 databases)**

**1. PubMed**

((((((((((((Knee Osteoarthritides) OR Knee Osteoarthritis) OR Osteoarthritides, Knee) OR Osteoarthritis Of Knee) OR Knee, Osteoarthritis Of) OR Knees, Osteoarthritis Of) OR Osteoarthritis Of Knees)) OR "Osteoarthritis, Knee"[Mesh])) AND ((((((((((((((((Acupuncture) OR acupuncture therapy) OR Therapy, Acupuncture) OR Treatment, Acupuncture) OR Acupuncture Treatment) OR Acupotomy) OR Acupotomies) OR Acupuncture Point) OR Point, Acupuncture) OR Acupoint) OR Electroacupuncture) OR Warm Acupuncture) OR Needle) OR Warm Needle)) OR ("Acupuncture"[Mesh] OR "Acupuncture Therapy"[Mesh] OR "Acupuncture Points"[Mesh]))) AND (((((((((((((((Hyaluronic Acid) OR Acid, Hyaluronic) OR Amo Vitrax) OR Vitrax, Amo) OR Biolon) OR Etamucine) OR Hyaluronan) OR Hyvisc) OR Luronit) OR Sodium Hyaluronate) OR Hyaluronate, Sodium) OR Amvisc) OR Healon)) OR "Hyaluronic Acid"[Mesh])) AND ((((((((((randomized controlled trial) OR controlled clinical trial) OR randomized) OR placebo) OR ramdomly) OR groups) OR Trial) OR random)) OR "Randomized Controlled Trials as Topic"[Mesh])

**2. The Cochrane Library**

ID Search Hits

#1 MeSH descriptor: [Osteoarthritis, Knee] explode all trees 3598

#2 (knee):ti,ab,kw 25550

#3 (Knee Joint):ti,ab,kw (Word variations have been searched) 9386

#4 (Joint, Knee):ti,ab,kw (Word variations have been searched) 9386

#5 (Knee Joints):ti,ab,kw (Word variations have been searched) 9383

#6 (Osteoarthritis):ti,ab,kw (Word variations have been searched) 15505

#7 (Osteoarthritides):ti,ab,kw (Word variations have been searched) 0

#8 (Osteoarthrosis):ti,ab,kw (Word variations have been searched) 467

#9 (Osteoarthroses):ti,ab,kw (Word variations have been searched) 2

#10 (Arthritis, Degenerative):ti,ab,kw (Word variations have been searched) 230

#11 (Arthritides, Degenerative):ti,ab,kw (Word variations have been searched) 0

#12 (Degenerative Arthritides):ti,ab,kw (Word variations have been searched) 0

#13 (Degenerative Arthritis):ti,ab,kw (Word variations have been searched) 230

#14 (Osteoarthrosis Deformans):ti,ab,kw (Word variations have been searched) 5

#15 #2 or #3 or #4 or #5 25630

#16 #6 or #7 or #8 or #9 or #10 or #11 or #12 or #13 or #14 15739

#17 #15 and #16 10525

#18 #17 or #1 10525

#19 (Hyaluronic Acid):ti,ab,kw (Word variations have been searched) 2791

#20 (Acid, Hyaluronic):ti,ab,kw (Word variations have been searched) 2791

#21 (Amo Vitrax):ti,ab,kw (Word variations have been searched) 1

#22 (Biolon):ti,ab,kw (Word variations have been searched) 3

#23 (Etamucine):ti,ab,kw (Word variations have been searched) 0

#24 (Hyaluronan):ti,ab,kw (Word variations have been searched) 433

#25 (Hyvisc):ti,ab,kw (Word variations have been searched) 0

#26 (Luronit):ti,ab,kw (Word variations have been searched) 0

#27 (Sodium Hyaluronate):ti,ab,kw (Word variations have been searched) 929

#28 (Amvisc):ti,ab,kw (Word variations have been searched) 18

#29 (Healon):ti,ab,kw (Word variations have been searched) 142

#30 #19or#20or#21or#22or#23or#24or#25or#26or#27or#28or#29 3343

#31 MeSH descriptor: [Acupuncture] explode all trees 141

#32 (acupuncture):ti (Word variations have been searched) 9867

#33 MeSH descriptor: [Acupuncture Therapy] explode all trees 4342

#34 MeSH descriptor: [Acupuncture Points] explode all trees 1835

#35 (Acupuncture Point):ti (Word variations have been searched) 626

#36 (Point, Acupuncture):ti (Word variations have been searched) 626

#37 (Acupoint):ti (Word variations have been searched) 1801

#38 (Acupotomy):ti (Word variations have been searched) 54

#39 (Acupotomies):ti (Word variations have been searched) 54

#40 (Electroacupuncture):ti (Word variations have been searched) 1498

#41 (Needle acupuncture):ti (Word variations have been searched) 299

#42 (warm Needle):ti (Word variations have been searched) 156

#43 #31or#32or#33or#34or#35or#36or#37or#38or#39or#40or#41or#42 13676

#44 #18and#30and#43 22

**3. EMBASE**

No. Query Results Results Date

#45. #10 AND #20 AND #35 AND #44 37 29 Aug 2019

#44. #36 OR #37 OR #38 OR #39 OR #40 OR #41 OR #42 OR#43 4,171,431 29 Aug 2019

#43. 'random':ab,ti 308,332 29 Aug 2019

#42. 'trial':ab,ti 809,961 29 Aug 2019

#41. 'groups':ab,ti 2,801,191 29 Aug 2019

#40. 'ramdomly':ab,ti 60 29 Aug 2019

#39. 'placebo':ab,ti 296,348 29 Aug 2019

#38. 'randomized':ab,ti 696,941 29 Aug 2019

#37. 'controlled clinical trial'/exp 732,647 29 Aug 2019

#36. 'randomized controlled trial'/exp 563,613 29 Aug 2019

#35. #21 OR #22 OR #23 OR #24 OR #25 OR #26 OR #27 OR 43,451 29 Aug 2019

#28 OR #29 OR #30 OR #31 OR #32 OR #33 OR #34

#34. 'healon':ab,ti 516 29 Aug 2019

#33. 'amvisc':ab,ti 36 29 Aug 2019

#32. 'hyaluronate sodium':ab,ti 73 29 Aug 2019

#31. 'hyaluronate, sodium':ab,ti 73 29 Aug 2019

#30. 'sodium hyaluronate':ab,ti 2,588 29 Aug 2019

#29. 'luronit':ab,ti 2 29 Aug 2019

#28. 'hyvisc':ab,ti 4 29 Aug 2019

#27. 'hyaluronan':ab,ti 10,734 29 Aug 2019

#26. 'etamucine':ab,ti 0 29Aug 2019

#25. 'biolon':ab,ti 27 29 Aug 2019

#24. 'vitrax, amo':ab,ti 0 29Aug 2019

#23. 'amo vitrax':ab,ti 4 29 Aug 2019

#22. 'acid, hyaluronic':ab,ti 67 29 Aug 2019

#21. 'hyaluronic acid'/exp 41,203 29 Aug 2019

#20. #11 OR #13 OR #14 OR #15 OR #16 OR #17 OR #18 OR#19 46,111 29 Aug 2019

#19. 'electroacupuncture':ab,ti 5,012 29 Aug 2019

#18. 'acupoint':ab,ti 3,283 29 Aug 2019

#17. 'acupotomies':ab,ti 0 29 Aug 2019

#16. 'acupotomy':ab,ti 75 29 Aug 2019

#15. 'needle acupuncture':ab,ti 295 29 Aug 2019

#14. 'acupuncture needle':ab,ti 535 29 Aug 2019

#13. 'warm needle':ab,ti 30 29 Aug 2019

#12. 'needle':ab,ti 148,627 29 Aug 2019

#11. 'acupuncture'/exp OR 'acupuncture point'/exp 45,669 29 Aug 2019

#10. #1 OR #2 OR #3 OR #4 OR #5 OR #6 OR #7 OR #9 31,567 29 Aug 2019

#9. 'osteoarthritides, knee':ab,ti 0 29 Aug 2019

#8. 'knee osteoarthritides':ab,ti 0 29 Aug 2019

#7. 'osteoarthritis of knees':ab,ti 27 29 Aug 2019

#6. 'knees, osteoarthritis of':ab,ti 1 29 Aug 2019

#5. 'knee, osteoarthritis of':ab,ti 74 29 Aug 2019

#4. 'osteoarthritis of knee':ab,ti 282 29 Aug 2019

#3. 'osteoarthritis, knee':ab,ti 557 29 Aug 2019

#2. 'knee osteoarthritides':ab,ti 0 29 Aug 2019

#1. 'knee osteoarthritis'/exp 31,417 29 Aug 2019

**4. China National Knowledge Infrastructure (CNKI)**

AB=(‘膝骨关节炎’+‘KOA’+'骨痹'+'膝关节骨性关节炎')*('针灸'+'温针'+'针刺'+'电针'+'针刀')*('玻璃酸钠'+'透明质酸钠')*('随机对照'+'随机分配'+'随机'+'rct')

**5. Wanfang Database**

主题:((“膝骨关节炎”+“KOA”+“骨痹”+“膝关节骨性关节炎”)*(“针灸”+“温针”+“针刺”+“电针”+“针刀”)*(“玻璃酸钠”+“透明质酸钠”)*(“随机对照”+“随机分配”+“RCT”+“随机”))

**6. Chinese Scientific Journal Database (VIP database)**

R=((“膝骨关节炎”+“KOA”+“骨痹”+“膝关节骨性关节炎”)*(“针灸”+“温针”+“针刺”+“电针”+“针刀”)*(“玻璃酸钠”+“透明质酸钠”)*(“随机对照”+“随机分配”+“随机”+“RCT”))

**7. Chinese Biomedical Literature Database (Sinomed)**

序号 检索表达式 命中文献数

1) "骨关节炎, 膝"[不加权:扩展] 17634

2) "膝骨关节炎"[摘要:智能] OR "膝关节骨性关节炎"[摘要:智能]

OR "膝骨痹"[摘要:智能] OR "KOA"[摘要:智能] 13133

3) (#2) AND (#1) 11688

4) (((("针刺疗法"[不加权:扩展]) OR "针刺穴位"[不加权:扩展]) OR "针刺"[不加权:扩展])

OR "电针"[不加权:扩展]) OR "温针疗法"[不加权:扩展] 70638

5) "针灸疗法"[不加权:扩展] 141087

6) "针刺疗法"[摘要:智能] OR "针刺"[摘要:智能] OR "针刺穴位"[摘要:智能]

OR "电针"[摘要:智能] OR "温针"[摘要:智能] OR "针灸"[摘要:智能] OR "针刀"[摘要:智能] 131783

7) (#6) OR (#5) OR (#4) 203445

8) "玻璃酸钠"[摘要:智能] OR "透明质酸钠"[摘要:智能] 6381

9) ("随机对照试验"[不加权:扩展]) OR "随机分配"[不加权:扩展] 479848

10) "随机对照"[摘要:智能] OR "随机分配"[摘要:智能] OR "RCT"[摘要:智能] 48263

11) (#10) OR (#9) 506861

12) (#11) AND (#8) AND (#7) AND (#3) 105

13) "随机"[常用字段:智能] 1405139

14) (#13) OR (#10) OR (#9) 1406029

15) (#14) AND (#8) AND (#7) AND (#3) 235

**Appendix 2 Acupuncture points**

| Source | Method | Acupuncture points of combination therapy group（international code） |
| --- | --- | --- |
| Chen Ying  (2015) | AT | - |
| Gao Yan  （2017） | WA | Main acupoints: Liangqiu(ST34), Ashipoints of lesion side, Xuehai(SP10),  Yanglingquan(GB34), Yinglingquan (SP9);  Matching acupoints: Weizhong(BL40), Fengshi(GB31),Huantiao(GB30),  Fengmen(BL12),Fengchi(GB20), Shenshu(BL23), Guanyuan(BL26), Zusanli(ST36),  Houxi(SI3),Taixi(KI03),Geshu(BL17),Gongsun(SP04), Zhongwan(RN12) |
| Han Dongming  (2016) | WA | Main acupoints: Neixiyan(Ex-LE 4),Yanglingquan(GB34), Dubi(ST35),  Weizhong(BL40), Zusanli(ST36),  Matching acupoints: Shenshu(BL23), Geshu(BL17), Yinglingquan (SP9); |
| Hao Yifan  (2017) | MA | Main acupoints: Yanglingquan(GB34), Yinglingquan (SP9);  Weizhong(BL40), Dubi(ST35), Neixiyan(Ex-LE4), Heding(Ex-LE02)  Matching acupoints:Zusanli(ST36),Xuehai(SP10), Shenshu(BL23),  Taixi(KI03), Dìjī (SP08),Sanyinjiao(SP06) |
| He Haijun  (2016) | AT | - |
| Lv Li  (2017) | MA | Main acupoints:Waixiyan(EX-LE5),Neixiyan(Ex-LE4),Yanglingquan(GB34)  ,Heding(Ex-LE02),Xuehai(SP10),Liangqiu(ST34),Zusanli(ST36),  Kunlun(BL60),Xuanzhong(GB39) |
| Ren Jing  (2012) | WA | Main acupoints:Neixiyan(Ex-LE4), Waixiyan (EX-LE5),  Yanglingquan(GB34), |
| Ren Xiaogang  (2015) | AT | - |
| Tian Huijun  (2018) | WA | Main acupoints:Dubi(ST35), Neixiyan(Ex-LE4), Weizhong(BL40),  Zusanli(ST36), Yanglingquan(GB34),  Matching acupoints:Shenshu(BL23), Geshu(BL17), Yinglingquan (SP9); |
| Zhao Zhichao  (2018) | WA+AT | Neixiyan(Ex-LE4), Waixiyan (EX-LE5)，Heding(Ex-LE02)，  Yanglingquan(GB34), Xuehai(SP10), Liangqiu(ST34), Zusanli(ST36),  Kunlun(BL60),Xuanzhong(GB39)  - |
